# Is Neck Circumference an Indicator for Metabolic Complication of Childhood Obesity?

**DOI:** 10.3889/oamjms.2015.012

**Published:** 2015-01-19

**Authors:** Nayera E. Hassan, Abeer Atef, Sahar A. El-Masry, Amany Ibrahim, Muhammad Al-Tohamy, Enas Abdel Rasheed, Galal Ismail Ahmed Elashry

**Affiliations:** 1 *Biological Anthropology Department, National Research Centre, Cairo, Egypt (Affiliation ID 60014618)*; 2 *Department of Pediatrics, Diabetes Endocrine and Metabolism Pediatric Unit (DEMPU), New Children’s Hospital, Faculty of Medicine, Cairo University, Cairo, Egypt*; 3 *Clinical Pathology Department, National Research Centre, Cairo, Egypt (Affiliation ID 60014618)*

**Keywords:** Neck Circumference, Children, Obesity, blood pressure, lipid profile

## Abstract

**BACKGROUND:**

The possible role of neck circumference (NC) for screening childhood obesity and its complication is not well characterized.

**AIM:**

To assess NC and to explore its increase as risk factor with metabolic syndrome (MS) variables.

**METHODS:**

Cross sectional case-control study included 50 obese children (BMI ≥95^th^ percentile) and 50 healthy (BMI 15^th^-‹85^th^ percentile). All were subjected to clinical examination, measuring blood pressure (BP), body weight, height, NC, waist (WC) and hip (HC)., fasting blood glucose, insulin and lipid profile.

**RESULTS:**

MS was detected among 52% of obese participants, but not among controls (0%). Clinical parameters and most of the laboratory values were higher in subjects with MS than in non-metabolic subjects, with statistical significance only in blood pressure and triglycerides. Among obese without MS, NC showed significantly positive correlations with age, weight, height, WC, HC and negative with LDL. While among Obese with MS, NC showed significantly positive correlations with age, weight, height, BMI-SDS, WC, HC and DBP.

**CONCLUSION:**

NC can be considered as a good indicator and predictor for obesity, especially central obesity. However, NC has no relation with lipid profile or fasting blood sugar.

## Introduction

Obese persons may suffer from an increased mortality risk due to cardiovascular disorders related to either continuously impairment of their lipid profile, blood pressure and/or insulin resistance. The continuing increase in the number of obese children is alarming due to the potential risk of premature health problems [1, 2]. In Egypt, the study of Hassan et al [3] revealed that the prevalence of obesity, among school children, was 8%, where as the prevalence of overweight was 11%.

Childhood obesity has significant adverse health consequences, as it is associated with dyslipidaemia, hypertension, glucose intolerance and it predisposes to early CVS disease. These constellations of metabolic disturbances have been defined as Metabolic Syndrome (MS). The Metabolic Syndrome (MS) has become one of the most severe health problems of the 21^st^ century [4]. Moreover, obese children have a high likelihood of becoming obese adults [5], who have a lower treatment response than those who become obese in adulthood [6].

Vague [7] was the first to realize that different body morphology or types of fat distribution are related to the health risks associated with obesity. He used a neck skin fold in his index of masculine differentiation to assess upper-body fat distribution.

To date, a relatively large number of studies have been conducted, on children, to examine the associations between NC and varying health indicators (such as cardiovascular risk factors) e.g. Androutsos et al [8] in Greece; Ben-Noun & Laor [9] in Israel and Kurtoglu et al [10] in Turkey; and between NC and prehypertension, e.g. Guo et al [11] in China. However, limited scientific evidence has been established to determine whether or not NC measurement can serve as a useful tool for classifying childhood overweight/obesity. Reviewing literature, the current study is the first one; among Egyptian children; that examines the efficacy of NC measurement in classifying childhood obesity in relation to a criterion measure of metabolic syndrome in an independent sample of Egyptian children.

Therefore, the purposes of this study were to: 1) Determine NC in a group of obese children as compared to a group of healthy non-obese children; serving as controls. 2) Explore the risk factors of increased NC among obese children and other variables of metabolic syndrome, such as hypertension, abnormal lipid profile and presence of insulin resistance.

## Subjects and Methods

This study is a cross sectional case-control one, conducted on 50 obese subjects (27 males and 23 females); whose BMI was ≥95^th^ percentile for age and sex, based on the Egyptian Growth Reference Charts [12]. Their ages ranged between 7-12 years. These subjects were recruited from Diabetes Endocrine and Metabolism Pediatric Unit (DEMPU) at Children Hospital, Cairo University, during the period from April 2013 to January 2014. Cases were compared to 50 healthy children (25 male and 25 female), whose BMI ranged from 15^th^ to ‹85^th^ percentile, age and sex matched, who were included as controls. All subjects belonged to the same social class (low-middle). Children with chronic illness, identified syndromes, chromosomal defects or endocrine disorders causing obesity, or those on chronic use of glucocorticoids were excluded from the study.

All children were subjected to history taking, complete clinical examination, and blood pressure assessment, anthropometric assessment (body weight, height, neck circumference, waist and hip circumferences). Anthropometric measurements were attempted following the recommendations of International Biological Program [13]. Body height was measured to the nearest 0.1 cm using a fixed stadiometer and body weight was determined to the nearest 0.01 kg using a Seca Scale Balance, with the subject wearing minimal clothing and no shoes. Waist circumference was measured at the midpoint between the lower rib margin and the iliac crest with the subject standing at the end of normal expiration, hip circumference at the level of the iliac crest, neck circumference (NC) in the midway of the neck (between midcervical spine and midanterior neck), using non-stretchable plastic tape to the nearest 0.1 cm. In men with a laryngeal prominence (Adam’s apple), NC was measured just below the prominence. All circumferences were taken with the subjects standing upright, with the face directed forward and shoulders relaxed. The following adiposity indices were calculated: Body mass index (BMI): as weight (in kilograms) divided by height (in meters) squared and Waist/Hip ratio (cm/cm).

Morning blood glucose, serum insulin and lipid profile were measured after an overnight fasting. Plasma glucose was determined by the glucose oxidase method. Plasma insulin was measured using ELISA immunoassay (DRG Diagnostic Products Corporation, Los Angeles, CA). Blood concentrations of total cholesterol and triglycerides were estimated in serum using calorimetric assay kit produced by P. Z. cormay, Lublin, Poland. High-density lipoprotein-cholesterol (HDL-C) was determined in serum by using calorimetric assay kits produced by Stanbio laboratory, Boerne, Texas. Low-density lipoprotein-cholesterol (LDL-C) was calculated as follows:

LDL-C= Total cholesterol –Triglycerides/5+ HDL-C.

Metabolic syndrome was diagnosed according to the National Cholesterol Education Program Adult Treatment Panel III criteria [14], which modified with adjustment for fasting blood glucose; according to the recent American Diabetes Association definition for impaired fasting glucose (ATP III/updated ADA); by Pedrosa et al [15]. Thus, MS was considered if three or more of the following criteria were present:

1) Abdominal obesity (WC ≥ 90^th^ percentile for age and sex).
2) Fasting TG ≥ 110 mg/dl.
3) HDL ≤ 40 mg/dl.
4) Systolic/diastolic BP ≥ 90^th^ percentile for age, sex and height.
5) Fasting glucose ≥ 100 mg/dl.

### Statistical Analysis

Statistical analysis was performed using Statistical Package for Social Sciences (SPSS^®^) for Windows^®^ version 16.0. Normality of the data was verified using the Kolmogorov-Smirnov test, which revealed that all the variables were of normal distribution. Measured data was described as mean and standard deviation (for parametric variables), number and percentage (for categorical variables). Difference between two groups was measured using unpaired student’s t-test (for parametric variables). Association between variables was assessed using Pearson`s correlation coefficient (for parametric variables). P-value <0.05 was considered significant [16].

## Results

Insignificant sex differences were detected in both obese and control groups for most of the parameters under study, except for hip circumference, where females had significantly higher values. So, the analysis was completed without sex differentiation.

Comparison between anthropometric, clinical and laboratory parameters of the obese group with the control one, showed that the obese group had significantly higher values in all parameters than those of control group, except for height SDS and HDL; where obese group were insignificantly taller and had insignificant lower HDL-c than the control (Tables 1, 2).

**Table 1:** Comparison between obese and controls as regards age and anthropometric parameters.

Parameters	Control, n=50		Obese, n=50		t	P
	Mean	± SD	Mean	± SD		
Age (year)	9.32	1.73	9.92	1.83	-1.69	0.095
Anthropometric						
Wt. SDS	0.09	0.61	3.01	1.47	-12.99	0.000**
Ht. SDS	-0.70	0.60	-0.49	1.04	-1.21	0.229
BMI (kg/m^2^)	18.42	1.95	29.90	3.18	-21.75	0.000**
BMI SDS	0.78	0.74	3.11	0.59	-17.31	0.000**
Neck circumference (cm)	29.18	1.74	33.35	1.62	-12.39	0.000**
Waist circumference (cm)	58.18	5.32	88.67	8.24	-21.98	0.000**
Hip circumference (cm)	69.66	7.71	94.97	10.26	-13.95	0.000**
Waist/Hip (cm/cm; n <0.72)	0.83	0.05	0.93	0.06	-9.76	0.000**

Wt. SDS (weight standard deviation score), Ht. SDS (height standard deviation score); BMI SDS (body mass index standard deviation score); P-value <0.05 was considered significant.

**Table 2:** Comparison between clinical and laboratory parameters among obese and controls.

Parameters	Control n=50		Obese n=50		t	P
	Mean	±SD	Mean	±SD		
Clinical						
SBP (mmHg)	101.52	5.95	114.14	9.08	-8.22	0.000**
DBP (mmHg)	63.52	5.78	77.10	11.48	-7.47	0.000**
Laboratory Data						
LDL (mg/dl)	87.84	19.97	100.36	30.10	-3.02	0.003**
HDL (mg/dl)	43.46	11.10	41.94	10.35	.71	0.480
Cholesterol (mg/dl)	146.50	25.32	170.84	27.96	-4.56	0.000**
TG (mg/dl)	57.08	15.50	100.38	28.24	-9.51	0.000**
Glucose (mg/dl)	87.60	9.96	94.96	9.51	-3.78	0.000**
Insulin (µU/mL)	7.88	2.77	17.72	8.10	-8.13	0.000**
HOMA-IR	1.70	0.67	5.17	4.92	-8.28	0.000**

SBP, systolic blood pressure; DBP, diastolic blood pressure; HDL, high density lipoprotein; LDL, low density lipoprotein; TG, triglycerides; HOMA-IR, Homeostasis Model Assessment - Insulin Resistance; P-value, <0.05 was considered significant.

Concerning metabolic syndrome (MS); Using the ATP III/updated ADA by Pedrosa et al [15]; it was found that 26 (52%) of the obese participants had the MS; while none of the control group (0%) had MS. Frequencies of metabolic criteria among cases of metabolic syndrome were presented in (Table, 3).

**Table 3:** Frequencies of metabolic criteria among cases of metabolic syndrome.

Parameters	MS subjects N=26	
	N	%
Anthropometric		
Waist circumference (cm) ≥ 90^th^ percentile	26	100
Clinical Data		
SBP (mmHg) ≥ 90^th^ percentile	22	84.6
DBP (mmHg) ≥ 90^th^ percentile	21	80.8
Laboratory Data		
HDL-C ≤ 40 mg/dl	12	46.2
TG ≥ 110 mg/dl.	12	46.2
Fasting glucose ≥ 100 mg/dl	0	0.0

The prevalence of, increased waist circumference was 100%, high SBP was 84.6%, DBP was 80.8%, TG was 46.2, low HDL was 46.2% and that of impaired fasting glucose was 0%.

Comparing obese participants; with and without MS (Tables 4 & 5) revealed that obese subjects with MS had significantly higher values in SBP, DBP and triglycerides than obese without MS.

**Table 4:** Comparison between MS and non MS subjects in obese group concerning age and anthropometric parameters.

Parameters	Without MS (n= 24)		With MS (n= 26)		t	p
	Mean	± SD	Mean	± SD		
Age (year)	10.21	1.72	9.65	1.92	1.07	0.288
Anthropometric						
Weight (kg)	55.50	11.70	55.40	12.80	0.03	0.978
Wt. SDS	2.72	1.54	3.28	1.37	-1.37	0.177
Height (cm)	135.69	11.46	134.82	12.29	0.26	0.799
Ht. SDS	-0.32	1.00	-0.68	1.08	-1.21	0.231
BMI (kg/m^2^)	29.88	3.12	29.93	3.30	-0.06	0.951
BMI SDS	3.06	0.62	3.15	0.57	-0.57	0.572
Neck circumference (cm)	33.39	1.55	33.34	1.70	0.20	0.843
Waist circumference (cm)	88.75	8.83	88.60	7.83	0.07	0.948
Hip circumference (cm)	95.67	10.61	94.34	10.09	0.46	0.649
Waist/Hip ratio (cm/cm)	0.93	0.06	0.94	0.05	-0.62	0.542

Wt. SDS (weight standard deviation score); Ht. SDS (height standard deviation score); BMI SDS (body mass index standard deviation score).

**Table 5:** Comparison between MS and non MS subjects in total sample concerning clinical and laboratory data.

Parameters	Without MS (n=24)		With MS (n=26)		t	p
	Mean	±SD	Mean	±SD
Clinical						
SBP (mmHg)	107.92	5.69	119.88	7.77	-6.17	0.000**
DBP (mmHg)	70.83	6.70	82.88	12.01	-4.42	0.000**
Laboratory Data						
LDL (mg/dl)	105.38	17.41	101.46	38.81	0.47	0.644
HDL (mg/dl)	41.29	8.39	42.54	12.01	-0.42	0.675
Cholesterol (mg/dl)	176.46	23.60	165.65	31.01	1.39	0.170
TG (mg/dl)	92.25	21.79	107.88	31.68	-2.02	0.047*
Fasting Glucose (mg/dl)	93.29	9.26	96.50	9.66	-1.19	0.237
Fasting Insulin (µU/mL)	17.33	9.63	18.08	6.56	-0.32	0.749
HOMA-IR	4.04	2.38	4.31	1.64	-0.46	0.650

SBP systolic blood pressure; DBP diastolic blood pressure; HDL (high density lipoprotein; LDL = low density lipoprotein; TG = triglycerides; HOMA-IR (Homeostasis Model Assessment- Insulin Resistance).

However, there were insignificant differences between the 2 groups regarding all anthropometric measures under study, fasting blood sugar, insulin, HOMA and cholesterol (total HDl-C and LDL-C).

The correlations between neck circumference and other parameters; among obese participants without MS (Table, 6) have revealed significant positive correlations with age (r=o.62, p‹ 0.001), weight (r=0.54, p‹ 0.007), height (r=0.50, p‹ 0.012), waist and hip circumferences (r=0.61, p‹ 0.002; r=0.66, p ‹ 0.000 respectively) and LDL-C (r=0.44, p‹ 0.030). While among obese participants with MS (Table, 7), there were significant positive correlations between neck circumference and age (r=o.62, p‹ 0.001), weight (r=0.63, p‹ 0.001), height (r=0.71, p‹ 0.000), waist and hip circumferences (r=0.40, p‹ 0.041; r=0.47, p‹ 0.016 respectively) and diastolic blood pressure (r=0.45, p‹ 0.023). So, it was evident that the current study, illustrates the relationship between neck circumference and the different parameters of diagnostic criteria of metabolic syndrome.

**Table 6:** Correlation between neck circumference and different parameters of diagnostic criteria of metabolic syndrome among Non metabolic subjects.

Parameters	Without MS (n=24)	
	r	p-value
Age (year)	0.619	0.001**
Anthropometric		
Weight (kg)	0.535	0.007**
Wt. SDS	-0.118	0.584
Height (cm)	0.502	0.012*
Ht. SDS	0.044	0.839
BMI (kg/m^2^)	0.330	0.115
BMI SDS	-0.180	0.400
Waist circum (cm)	0.605	0.002**
Hip circum (cm)	0.661	0.000**
Waist/Hip (n <0.72)	-0.203	0.340
Clinical		
SBP (mmHg)	0.048	0.823
DBP (mmHg)	0.186	0.384
Laboratory Data		
LDL (mg/dl)	-0.444	0.030*
HDL (mg/dl)	-0.139	0.516
Cholesterol (mg/dl)	-0.221	0.299
TG (mg/dl)	0.314	0.135
Fasting Glucose (mg/dl)	-0.137	0.524
Fasting Insulin (µU/mL)	0.119	0.580
HOMA-IR	0.116	0.591

Wt. SDS, (weight standard deviation score); Ht. SDS (height standard deviation score); BMI SDS, (body mass index standard deviation score); SBP, systolic blood pressure; DBP, diastolic blood pressure; HDL, high density lipoprotein; LDL, low density lipoprotein; TG, triglycerides; HOMA-IR, (Homeostasis Model Assessment- Insulin Resistance).

**Table 7:** Correlation between neck circumference and different parameters of diagnostic criteria of metabolic syndrome among metabolic subjects.

Parameters	With MS (n=26)	
	r	p-value
Age (year)	0.623	0.001
Anthropometric		
Weight (kg)	0.631	0.001**
Wt. SDS	-0.087	0.674
Height (cm)	0.706	0.000**
Ht. SDS	0.138	0.500
BMI (kg/m^2^)	0.239	0.240
BMI SDS	0.403	0.041*
Waist circumference (cm)	0.465	0.017*
Hip circumference (cm)	0.466	0.016*
Waist/Hip (n <0.72)	-0.113	0.582
Clinical		
SBP (mmHg)	0.289	0.152
DBP (mmHg)	0.445	0.023*
Laboratory Data		
LDL (mg/dl)	0.122	0.551
HDL (mg/dl)	-0.120	0.559
Cholesterol (mg/dl)	0.056	0.787
TG (mg/dl)	-0.253	0.212
Fasting Glucose (mg/dl)	-0.377	0.058
Fasting Insulin (µU/mL)	0.219	0.283
HOMA-IR	0.113	0.583

Wt. SDS, (weight standard deviation score); Ht. SDS, (height standard deviation score); BMI SDS, (body mass index standard deviation score); SBP, systolic blood pressure; DBP, diastolic blood pressure; HDL, (high density lipoprotein; LDL, low density lipoprotein; TG, triglycerides; HOMA-IR, (Homeostasis Model Assessment- Insulin Resistance).

## Discussion

Obesity is known to be associated with inflammatory changes, insulin resistance and with hyperglycemic state [17]. Cardiovascular system (CVS) morbidity and mortality is associated with the classic risk factors namely dyslipidemia, hypertension and impaired glucose metabolism [18]. One of the complications of obesity is insulin resistance (IR) which; if persists; leads to glucotoxicity leading to chronic hyperglycemia and clinical diabetes [19]. Previous studies have been performed on obese subjects to explore the clinical significance of high normal fasting blood glucose (FBG). Insulin resistance (IR) has been implicated in the pathogenesis of metabolic syndrome, moreover obesity in children and adolescents is the most common feature associated with IR [20].

The current study showed a significant increase in NC in the obese group compared to the control group, hence it documents the relationship between NC and obesity. This agrees with the previous results of Yang et al. [21] and Lou et al. [22] in China; Androutsos et al. [8] in Greece and Nafiu et al. [23] and Kim et al. [24] in USA. All of the above-mentioned studies advocated the use of NC measurement, primarily based on its “practicality” for clinical settings; as it is easy/simple/inexpensive to use, unnecessary to remove upper clothes, and less susceptible to harsh weather than other measures (i.e., waist circumference measure).

In addition, the current study revealed that obese participants have statistically significant higher levels of SBP, DBP, LDL-C, total cholesterol, triglycerides, fasting blood glucose, insulin and HOMA-IR than the control group. Although HDL value was lower in the obese group compared to the control one, it was statistically insignificant. Such findings could be explained by the high intake of saturated fat and the low intake of dietary fiber as a general eating habit of the Egyptians; and mostly it is due to the increase consumption of fast food as a global phenomenon including Egypt. In concordance with the current study, Grandone et al [25]; in Italy; found that high normal FBG was associated with seven folds risk of presenting impaired glucose tolerance and insulin resistance among 323 obese children. In a more recent study, O’Malley et al. [26], reported a reduction in both insulin sensitivity and B cell function at increasing FBG in normoglycemic multiethnic obese youth, thus demonstrating that some deterioration of glucose homeostasis is already present in obese youth even in apparently normal FBG.

Searching within the criteria of metabolic syndrome (MS), the present study revealed that 26 obese participants (52% of obese subjects) fulfilled the criteria of MS according to ATP III/updated ADA by Pedrosa et al. [15], while none of the control group fulfilled these criteria. Among the obese participants with MS group, the prevalence of increased waist circumference was 100%, high SBP 84.6%, DBP 80.8%, TG was 46.2, low HDL was 46.2% and impaired fasting glucose was 0%. In comparison with the previous study of Hassan and her colleague [3], who recorded that the prevalence of MS among prepubertal children; aged 7 up to 11 years, was 45.5%., the prevalence of increased waist circumference, high SBP, DBP, TG, low HDL and impaired fasting glucose were 100%, 78.6%, 75%, 46.4%, 35.7% and 3.6% respectively. This means that there is an increase in the prevalence of MS and its criteria among Egyptian children. However, Pedrosa et al.[15]; in Portugal; have reported that the prevalence of MS was 15.8% among 82 children (14 overweight and 68 obese) aged 7-9 years.

In the current study, obese subjects with MS had significantly higher values in SBP, DBP and triglycerides than those without MS. However, there were insignificant differences between the two groups, regarding all anthropometric measures under study; fasting blood sugar, insulin, HOMA and cholesterol (total, HDl-C and LDL-C). This agrees with the previous studies of Eapen et al., [27] in Emirates; López-Capapé et al.,[20] in Spain; Johnson et al., [28] in USA and Invitti et al., [29] for Caucasians. Pedrosa et al., [15], in Portugal; have found that BP and TG were significantly higher in subjects with MS than those of subjects without MS. In contrary with the current study, Pedrosa et al., [15], also reported significantly higher values of BMI and WC among subjects with MS than those without MS.

In the current study, although NC was not statistically different between the metabolic and the non-metabolic syndrome groups, NC showed significantly positive correlations with age, body weight, height, waist and hip circumferences among obese participants with or without MS, in addition to BMI SDS and DBP among those with MS. The positive correlation between NC and DBP indicates the strong relationship between obesity and hypertension. This finding is in agreement with that of Kurtoglu et al [10], in Turkey, and Nafiu et al., [30] in USA; who found that, the increased NC and BMI were associated with elevated BP in children. Yang et al [2] and Lou et al. [22]; in China; also concluded that NC was positively related with age, BMI, waist circumference and metabolic syndrome. Hence, it can be concluded that NC has a positive correlation with central obesity (WC) and hypertension (DBP) in the studied age group.

These concerns about the effects of obesity reinforce the need for the prevention and treatment of this condition in childhood. The importance of changing the lifestyle of these children should be emphasized, especially with regard to eating habits and the practice of regular physical activity.

In conclusion, neck circumference is a good predictor for obesity. It has highly significant positive correlations with some elements of MS criteria as body weight, waist circumference and DBP. So, NC can be considered as a good indicator and predictor for central obesity and hypertension, which are from the criteria of MS among children.
